# Modeling COVID-19 transmission between age groups in the United States considering virus mutations, vaccinations, and reinfection

**DOI:** 10.1038/s41598-022-21559-9

**Published:** 2022-11-22

**Authors:** Jyotirmoy Roy, Samuel M. Heath, Shiyan Wang, Doraiswami Ramkrishna

**Affiliations:** 1 Department of Chemical Engineering, Indian Institute of Technology Bombay, Mumbai, 400076 India; 2 Charles D. Davidson School of Chemical Engineering, Purdue University, West Lafayette, IN 47907 USA; 3 Present Address: Department of Biomedical Engineering, University of Michigan, Ann Arbor, MI 48109 USA; 4 Present Address: Department of Mechanical Engineering, Massachusetts Institute of Technology, Cambridge, MA 02139 USA

**Keywords:** Computational biophysics, Epidemiology

## Abstract

The in-depth understanding of the dynamics of COVID-19 transmission among different age groups is of great interest for governments and health authorities so that strategies can be devised to reduce the pandemic’s detrimental effects. We developed the SIRDV-Virulence (Susceptible-Infected-Recovered-Dead-Vaccinated-Virulence) epidemiological model based on a population balance equation to study the effects virus mutants, vaccination strategies, ‘Anti/Non Vaxxer’ proportions, and reinfection rates to provide methods to mitigate COVID-19 transmission among the United States population. Based on publicly available data, we obtain the key parameters governing the spread of the pandemic. The results show that a large fraction of infected cases comes from the adult and children populations in the presence of a highly infectious COVID-19 mutant. Given the situation at the end of July 2021, the results show that prioritizing children and adult vaccinations over that of seniors can contain the spread of the active cases, thereby preventing the healthcare system from being overwhelmed and minimizing subsequent deaths. The model suggests that the only option to curb the effects of this pandemic is to reduce the population of unvaccinated individuals. A higher fraction of ‘Anti/Non-vaxxers’ and a higher reinfection rate can both independently lead to the resurgence of the pandemic.

## Introduction

The coronavirus disease of 2019 (COVID-19) pandemic has brought global devastation since its inception in Wuhan, China in December 2019^1^. One challenging aspect of analyzing and predicting the COVID-19 pandemic is that its dynamics are continuously changing as a result of the continuous mutation of the severe acute respiratory syndrome coronavirus 2 (SARS-CoV-2), the virus which causes COVID-19^2^. Often, SARS-CoV-2 mutations can increase the transmission rate of the virus^3^ and decrease the vaccine efficacy^2^$$^,$$^4^$$^,$$^5^, which can create a possibility of resurgence of the pandemic^6^. Since their peaks in early January 2021, the COVID-19 cases and deaths had markedly declined, due in part to the increased vaccination coverage^7^. However, during June 19–July 23, 2021, COVID-19 cases increased approximately 30$${\%}$$ in the United States, followed by increases in hospitalizations and deaths^7^, driven by the highly transmissible B.1.617.2 (Delta) SARS-CoV-2 variant.

The Delta variant is more than two times as transmissible as the strains circulating at the start of the pandemic^8^ and has caused large, rapid increases in infections, putting pressure on local and regional health care systems^8^. Strains on critical care capacity can increase COVID-19 mortality^9,10^ while decreasing the availability and use of health care resources for non-COVID-19 related medical care^11,12^. Therefore, it is important to predict the timing and magnitude of peaks for active infections so that medical personnel are prepared and the government can introduce appropriate and informed policy decisions.

Despite widespread availability of vaccines and evidence that they markedly reduced hospitalizations and deaths in the United States^13^, vaccine uptake decreased nationally starting in May 2021. By the end of July, there was a wide variation in vaccination coverage by state (33.9${\%}$-67.2${\%}$) and by county (8.8${\%}$-89.0${\%}$)^14^. Unvaccinated and immunocompromised individuals^15^ remained at substantial risk for COVID-19-related infection, severe illness, and death, especially in areas with high SARS-CoV-2 transmission rates^16^. With a COVID-19 vaccine acceptance of only 67${\%}$^17^ along with increasing anti-vaccination rhetoric towards the COVID-19 vaccines in the US, there is concern that the effects of the SARS-CoV-2 mutations could be exacerbated if a significant proportion of the US population remains unvaccinated. While the government has focused on reducing the spread of misinformation and addressing the underlying concerns of the anti-vaxxer community^18^, it is important to provide a quantitative understanding of the effect of ‘Anti/Non-Vaxxers’ on the dynamics of COVID-19 transmission.

While our study does not consider the effect of changing immunity as a result of the time interval between inoculations, this has been studied by other authors. Krause et al.^19^ and Moghadas et al.^20^ discuss the significance of the timing of multiple doses of the COVID-19 vaccine and the effect of a delayed second dose on vaccine dynamics. Additionally, although the present study focuses on the effects of vaccinations, non-pharmaceutical interventions (NPIs) (contact tracing, testing, social distancing, wearing masks, sheltering in place, etc.) are also important in controlling the spread of COVID-19^21^. This is well supported by the evidence that South Korea^22^ implemented aggressive contact tracing and testing policies, reducing the detrimental effects of the outbreak. Similar results show that stay-at-home orders and face-mask wearing have a positive impact on decreasing the number of reported cases^23^. Yagan et al. demonstrated how their multiple-strain transmission model assess the effectiveness of mask-wearing in limiting the spread of COVID-19^24^.

Due to the possibility that recovered individuals can be reinfected by the SARS-CoV-2 virus, numerous studies have been carried out to quantify the effects of waning immunity and reinfection on COVID-19 dynamics. From January 1 through April 2021, 10,262 breakthrough infections were reported to the CDC in the US^25^. A less transmissible variant of COVID-19 which shows partial immune-escape can provoke a wave of infection until control measures are further tightened^26^. The Susceptible-Infected-Recovered-Susceptible (SIRS) model described in Rubin et al.^27^ takes into account the effect of partial escape to study the effect of reinfection in COVID-19 dynamics. Studies conducted by Ehrhardt et al.^28^ show that individuals who previously had COVID-19 immunity (recovered or vaccinated), may lose their immunity if the level of antibodies reaches a certain critical level.

Another element of primary concern is the varying dynamics among different age groups^29^. Age-related differences in COVID-19 responsiveness and tolerance have been identified, and it is known that elderly individuals tend to have worse clinical outcomes than younger individuals^30^. Previous studies show that older COVID-19 patients are at an increased risk of death^31–34^. Chikina et al. have developed a Susceptible-Infected-Recovered (SIR) epidemic model integrating known age-contact patterns for the US to model the effect of age-targeted mitigation strategies for a COVID-19-like epidemic^35^. Strict age-targeted mitigation strategies have the potential to greatly reduce mortality and ICU utilization. Another study reaffirmed that COVID-19 epidemic processes have had distinctive dynamic patterns among age and gender groups^36^. Accordingly, age-targeted vaccine strategies need to be developed to minimize COVID-19-related infections and deaths.

While the present study assumes spatially homogeneous age group populations across the US, it should be noted that in reality, COVID-19 dynamics vary from state to state. These effects are studied by Thomas et al.^37^, who shows that spatial heterogeneity can produce significant differences in social exposures to those with COVID-19. which can stress health care systems in ways that cannot be predicted by standard Susceptible-Infected-Recovered (SIR)-like models. Similarly, Zhong et al.^23^ show that the basic reproduction number varies greatly across the US.

**Figure 1:**
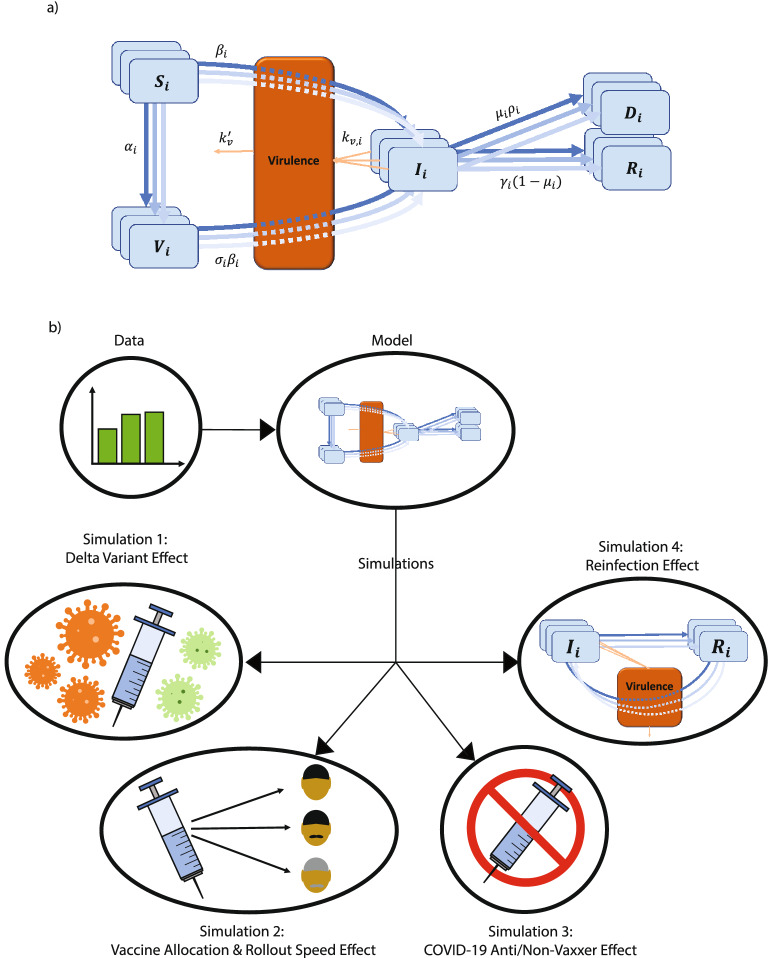
A schematic of the SIRDV-Virulence (Susceptible-Infected-Recovered-Dead-Vaccinated -Virulence) model and overall process of the study. (**a**) An SIRDV-Virulence model (Susceptible-Infected-Recovered-Dead-Vaccinated-Virulence) to predict the transmission of COVID-19 in the United States, in which the members of three age groups (children, adults, and seniors) move to compartments (blue) at rates influenced by the parameters adjacent to the inter-compartmental arrows (blue). Infected members of each age group contribute to the growth of a single virulence parameter (orange), which can infect both susceptible, ${S_i}$, and vaccinated, ${V_i}$, individuals. (**b**) Data is fed to the compartmental model to fit its parameters and used to run simulations to predict future scenarios: (1) the effect of the Delta variant, (2) the effect of changing the vaccination allocation and roll-out speed, (3) the effect of the proportion of COVID-19 Anti/Non-Vaxxers, and (4) the effect of reinfection.

In this work, we develop a novel compartmental model to predict COVID-19 infections and deaths among three different age groups. To simulate the interaction within and between the age groups, this model uses the infection brought by the virulence environment^38^, derived using population balance equations^39,40^. We first fit the model to COVID-19 cases, death, and vaccination data from January to July 2021. The fitted model is then used to make predictions starting in August 2021, focusing on four simulations, as illustrated in Fig. 1b. First, we evaluate how an increased transmission rate and vaccine inefficacy associated with the Delta variant can change the dynamics of the pandemic in the US. Second, we study the effect of changing the vaccine rollout speed and distribution to gain insight into an optimum vaccine distribution strategy to minimize COVID-19 infections and mortality. Third, we study the effect of changing the proportion of Anti/Non-Vaxxers in the US. Fourth, we study how incorporating reinfection into our model can change its predictions.

## Results

###  Parametric model fitting across multiple time periods

**Figure 2:**
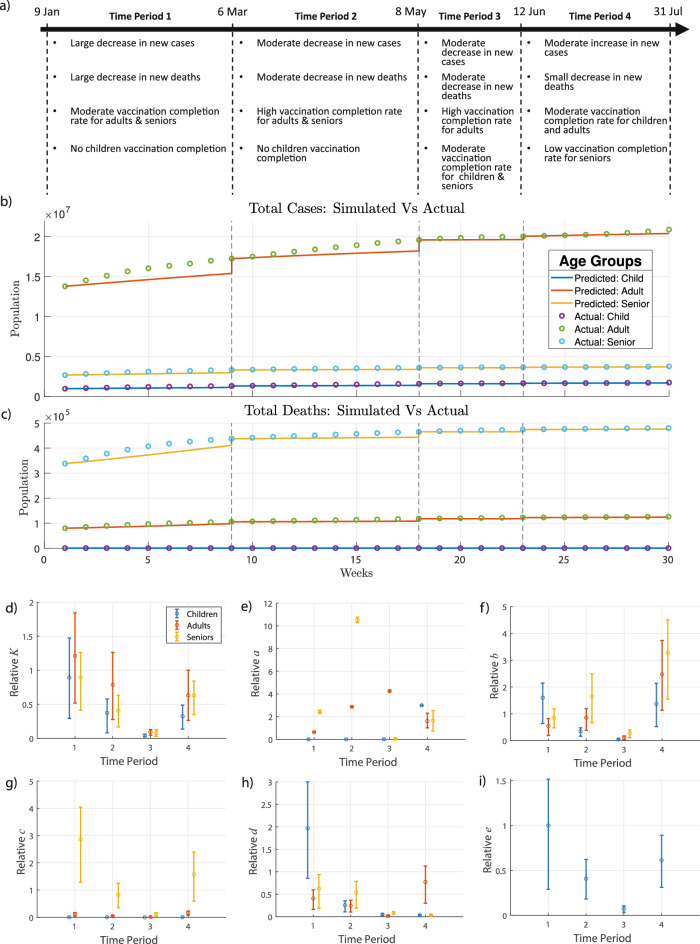
SIRDV-Virulence (Susceptible-Infected-Recovered-Dead-Vaccinated-Virulence) Model Fitting. (**a**) COVID-19 data are divided over four time periods based on the changing characteristics of the virus and vaccination dynamics. The fitted model is plotted against the data during each time period for (**b**) cumulative weekly cases, (**c**) cumulative weekly deaths, and cumulative weekly vaccinations (shown in the supplemental material). Fitted dimensionless parameters are shown for each age group (i=1-children,2-adults,3-seniors) during each of the four time periods: (**d**) viral transmission $K_i$, (**e**) vaccination rate $a_i$, (**f**) recovery rate $b_i$, (**g**) death rate $c_i$, (**h**) viral load $d_i$, and (**i**) life span of virus *e*. In (**d**–**i**), the mean is the central point, and the error bars represent the $25\mathrm{th}$ and $75\mathrm{th}$ percentile values from Monte Carlo fitting simulations; relative parameters are displayed, where each data point is relative to the average of the respective parameter values of the three age groups for the first time period.

Since the vaccine dynamics and effect of mutant variants varied from January to July of 2021, four time periods were considered for fitting, and the 16 parameters ($K_i,~a_i,~b_i,~c_i,~d_i,~e$ with *i* = 1-—children, 2—adults, 3—seniors) were obtained for each time period (see Fig. 2a). Three data sets were used for model fitting: COVID-19 Weekly Cases by Age, COVID-19 Weekly Deaths by Age, and COVID-19 Vaccinations by Age. The first time period considered was from January 9, 2021 to March 6, 2021. During this period, the most transmissible strain of COVID-19 present was the alpha variant^41^. The children were not vaccinated during this time period, but adults and seniors were. In the second period, from March 6 to May 8, 2021, it was assumed that the most transmissible COVID-19 strain present was the Delta strain since the CDC reports the introduction of the Delta variant in the US in early March of 2021^42^. During this period the adults and seniors were vaccinated and the children were not. During the third time period, May 8–June 12, 2021, all three age groups were vaccinated. The alpha and Delta variants were considered the most dominant strains, and the Delta variant was beginning to contribute to a significant proportion of recorded cases^43^. There was a moderate decrease in new cases and deaths during this period as seen in Fig. 2b, c. During the last period, June 12–July 31, 2021, the proportion of cases due to the Delta variant increased. During this period, the vaccination rates were higher for children as compared to adults and seniors (see Fig. 2e). In summary, 4 sets of 16 parameters were obtained across the four time periods.

The fitted dimensionless parameters have properly reflected the dynamics of COVID-19 transmissibility during these four periods. The value of the relative transmission rate, $K_i$, shows a decreasing trend for all three age groups during periods 1–3. However, as the delta became dominant, there was a sudden increase in the values of *K* for all age groups in period 4 (Fig. 2d).

The values of the vaccination parameter, $a_i$, correctly captured the vaccination dynamics in the US during the fitted time periods. During the first two time periods, $a_i$ was higher for the senior age group ($i=3$), which is consistent with prioritizing senior vaccination. In the last two periods (Fig. 2e), the children and adult age group, on average, have a higher vaccination rate than the senior age group.

The recovery rates for all three age groups show an increasing trend with time (Fig. 2f). This is likely due to the fact that a higher fraction of the population is gaining immunity through vaccinations during the fitted time periods. However, there is an increase in the mortality rate of the senior age group from the third to the fourth time period, indicated by a high value of *c* in Fig. 2g, which could be due to the Delta variant effect. Relative to the senior age group, the changes in mortality rates of children and adults are negligible, even during the last period when the Delta variant effect is visible in the population. Children contribute most to viral load in the first time period, while seniors contribute most during the second and third time period, and adults contributes most during the last period as indicated by the values of $d_i$ in Fig. 2h. The dimensionless number *e* shows a decreasing trend followed by a sharp increase in the last time period as seen in Fig. 2i. This indicates that the time to infect an individual times the viral death rate increases when the Delta variant effect is dominant.

**Figure 3:**
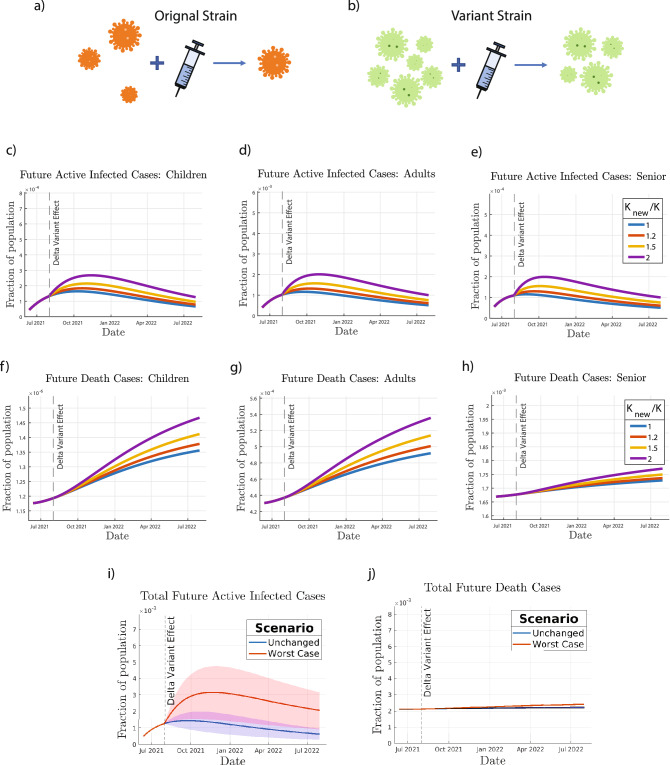
Effect of mutation on COVID-19 transmission. The mutant variant is modeled by assuming an increase in infection rate *K*, and an increase in vaccine inefficacy $\sigma$ and by comparing the (**a**) original and (**b**) variant strain, shown pictorially by a higher concentration of virus particles and a higher proportion of particles after vaccination. Future predictions of infected cases and deaths are simulated for (**c**, **f**) children, (**d**, **g**) adults, and (**e**, **h**) seniors at increasing relative infection rates (relative to *K* from the fourth fitted time period) and a constant $\sigma$ of 0.05. The worst case scenario (relative *K* of 2 and $\sigma$ of 0.2) is simulated, resulting in the predictions of (**i**) total future active infected cases and (**j**) total future deaths, where the shaded regions show the error introduced using the $25\mathrm{th}$ and $75\mathrm{th}$ percentiles (from Monte Carlo fitting simulations) of the relative *K* values.

In the following sections, we will discuss the simulated predictions considering four important factors: (1) effect of the Delta variant, (2) vaccine optimization, (3) effect of Anti/Non-Vaxxers, and (4) effect of reinfection.

### Effect of mutation on transmissibility

Mutation of SARS-CoV-2 has been largely responsible for the increased transmissibility due to an increased infection rate and reduced vaccine efficacy^44^. During the summer of 2021, the Delta variant was responsible for almost all recorded COVID-19 cases^45^. Therefore, it was important to study the effects of changes in transmissibility, $K_i$, and vaccine inefficacy, $\sigma$.

First, we study the variation in $K_i$ while keeping $\sigma$ constant. The simulated values of infection rate, $K_i$, were taken to be 1, 1.2, 1.5 and 2 times the $K_i$ values from the fourth fitted time period to take into account the effect of original strain and variants (Fig. 3a, b). For all age groups, the number of active cases increases with increasing $K_i$. Specifically, for a two-fold increase in $K_i$, the number of active cases at the peak of the pandemic increases by around 1.5–2 times for all age groups (Fig. 3c–e). In addition, a higher infection rate delays the time when the infection reaches its peak value, with differing peak infection times for each age group. Similarly, the total number of deaths increases with an increase in the value of $K_i$ for all age groups (Fig. 3f–h). The effect is more pronounced in children and adults as compared to seniors, which could be due to a larger fraction of unvaccinated children and adults, relative to the senior population. For a two fold increase in $K_i$, the total number of deaths increases by approximately 8$\%$ in children and 10$\%$ in adults, and only 2$\%$ in seniors.

For the total US population (excluding ages 12 and under), a comparison was made between two extreme scenarios (Fig. 3i, j): no change in fitted parameters (from the fourth fitted time period) and the worst case scenario. In the worst case scenario, the relative dimensionless infection rate is doubled, and the vaccine inefficacy is increased from 0.05 to 0.2. While the total number of deaths is not significantly affected in the worst case scenario, the peak active infected cases increases by almost 2.5 times. Further simulation scenarios can be found in the supplemental material.

### Vaccination optimization strategy

Optimization of vaccine distribution strategies among different age groups remains critical^46^. Specifically, it is necessary to study the effect of varying the vaccination rates and vaccination prioritization among each of the three age groups. Our study modeled the resulting completed vaccinations, cumulative cases, and cumulative deaths over a future time period as the vaccination parameters were varied.

We first determined the practical range for the dimensionless vaccination parameter, $a_i$, of each age group. To do this, we assumed that future vaccination rates in the US would not reach the rates that they had reached previously (considering both individual age groups and the entire population under study), given the majority of the senior and adult age groups had already been vaccinated by the initial date of the future simulation time period and peaks had already been reached for the completed vaccination rates in each age group.

**Figure 4:**
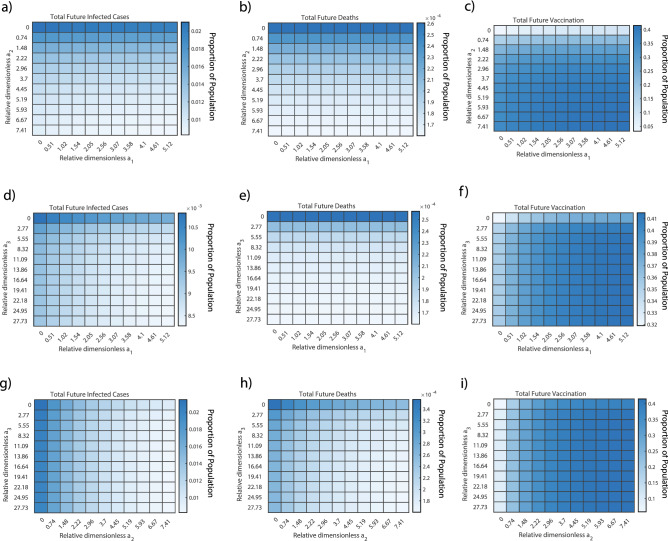
Vaccination Allocation Results. The effect of changing the vaccination allocation among each age group and vaccination roll-out speed is studied. (**a**–**c**) The senior vaccination parameter, $a_3$, is held at its maximum practical value, while the children and adult vaccination values, $a_1$ and $a_2$, respectively, are varied from 0 to their maximum practical values to predict the proportion of the United States population that will (**a**) become infected and (**b**) die as a result of COVID-19 from July 2021 to July 2022. (**c**) The total number of completed vaccinations from July 2021 to July 2022 as a result of the same parameter changes are shown for reference. (**d**–**f**) The adult vaccination parameter is held at its maximum value while the children and senior vaccination parameters are varied. (**g**–**i**) The children vaccination parameter is held at its maximum value while the adult and senior vaccination parameters are varied.

To study age group vaccination prioritization, a comparison was done using heat maps (Fig. 4). In general, the minimum infected cases and deaths and the maximum fraction of vaccinated population occur at the highest values of $a_i$ for these age groups, yet the dependence of vaccination rate in each age group is different. For instance, the future total infections and deaths, as well as total vaccinated fraction are more dependent on $a_2$ (adult age group) than on $a_1$ (children age group) as shown in Fig. 4a, b. This is likely because the fraction of the adult population is much higher than that of the children. Comparing the children and senior age groups, it was seen that the total death and infection were more strongly dependent on children than on seniors ($a_3$) (Fig. 4d, e). A similar comparison among the senior and adult age group showed that total death and infected cases is more dependent on adults than seniors (Fig. 4g, h).

The dependence of COVID-19 dynamics on adult and children vaccinations are likely due to the differences in completed vaccinations for each age group. A large fraction of seniors ($\sim$ 81.8%)^47^ had been fully vaccinated for COVID-19 by the end of July 2021. In comparison, only around 54.4% of the adult population was vaccinated by this time, while the percentage of children was about 34.4%^47^. Since a large fraction of the children and adult populations had yet to be vaccinated by the end of July 2021, a higher priority was needed to be given to these age groups over the senior age group for the future vaccine distribution, consistent with the strategy in the United States during that time^48^. For the vaccine distribution strategy, a higher priority to adult and children age groups over the senior age group was predicted to minimize total death and infections as the majority of the population in these two groups were more susceptible to the infection.

### Effect of anti/non-Vaxxer

The long-term effects of individuals unwilling and/or unable to receive the COVID-19 vaccination(s) was studied. Specifically, the effects of varying the proportion of COVID-19 ‘Anti/Non-Vaxxers’ in the susceptible population of each age group were observed to see how changes in the proportion of one age group could affect the number of deaths and cases of that same age group or other age groups.

**Figure 5:**
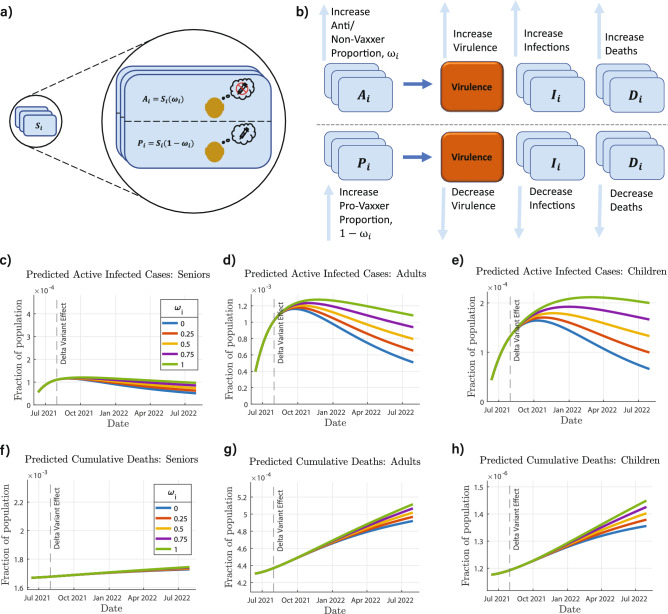
COVID-19 Anti/Non-Vaxxer Effect and the PAIRDV-Virulence ( ProVaxxer - AntiVaxxer - Infected - Recovered - Dead - Vaccinated - Virulence) Model. (**a**) The PAIRDV-Virulence model, a variation of the SIRDV-Virulence ( Susceptible - Infected - Recovered - Dead - Vaccinated - Virulence) model, is introduced, in which the susceptible population, $S_i$, is divided into a sub-population that will not be vaccinated, $A_i$, and a sub-population that will be vaccinated, $P_i$. (**b**) When the proportion of the susceptible population that will not be vaccinated, $\omega _i$, increases, the virulence, infections, and deaths increase (**c**–**h**). The predicted cases and deaths are shown for (**c**, **f**) seniors, (**d**, **g**) adults, and (**e**, **h**) children as a result of simultaneously changing $\omega _i$ for each age group.

To simulate this case, a modified compartmental model, PAIRDV-Virulence ( ProVaxxer-AntiVaxxer-Infected-Recovered-Dead-Vaccinated-Virulence), was developed that divided the Susceptible compartment into two new compartments: the COVID-19 ‘Anti/Non-Vaxxer’ compartment and the COVID-19 ‘Pro Vaxxer’ compartment, shown in Fig. 5a. The relationship between the Susceptible compartment of the SIRDV-Virulence ( Susceptible-Infected-Recovered-Dead-Vaccinated-Virulence) model and the COVID-19 ‘Anti/Non-Vaxxer’ and COVID-19 ‘Pro Vaxxer’ compartments of the PAIRDV-Virulence is $x_{P_i} = (1-\omega _i)x_{S_i}$ and $x_{A_i} = \omega _i x_{S_i}$, where $x_{P_i}=P_i/N$ represents the fraction of individuals of age group *i* in the COVID-19 ‘Pro Vaxxer’ compartment, and $x_{A_i}$ represents the fraction of individuals of age group *i* in the COVID-19 ‘Anti/Non-Vaxxer’ compartment. At the beginning of the time period of future simulations, $x_{S_i}$ is the sum of $x_{P_i}$ and $x_{A_i}$, and $\omega _i$ is the proportion of COVID-19 ‘Anti Vaxxers’ in the susceptible population of age group *i*.

Dimensionless equations were developed for the PAIRDV-Virulence model in order to conduct the future simulations:

1 $$\begin{aligned}&\frac{dx_{P,i}}{d\tau } = -x_{P,i}y\kappa _i-a_ix_{P,i}\;, \end{aligned}$$

2 $$\begin{aligned}&\frac{dx_{A,i}}{d\tau } = -x_{A,i}y\kappa _i\;, \end{aligned}$$

3 $$\begin{aligned}&\frac{dx_{I,i}}{d\tau } = x_{P,i}y\kappa _i+x_{A,i}y\kappa _i+\sigma x_{V,i}y\kappa _i-b_ix_{I,i}-c_ix_{I,i}\;, \end{aligned}$$

4 $$\begin{aligned}&\frac{dx_{R,i}}{d\tau } = b_ix_{I,i}\;, \end{aligned}$$

5 $$\begin{aligned}&\frac{dx_{D,i}}{d\tau } = c_ix_{I,i}\;, \end{aligned}$$

6 $$\begin{aligned}&\frac{dx_{V,i}}{d\tau } = a_ix_{P,i}-\sigma x_{V,i}y\kappa _i\;, \end{aligned}$$

7 $$\begin{aligned}&\frac{dy}{d\tau } = \sum ^{3}_{i=1}d_ix_{I,i}-ey\;, \end{aligned}$$

The PAIRDV-virulence model has the same parameters as the SIRDV-virulence, but one additional compartment. For COVID-19 ‘Anti/Non-Vaxxers’, the only way to exit the $x_{A,i}$ compartment is by COVID-19 infection whereas the COVID-19 ‘Pro Vaxxers’ can exit the $x_{P,i}$ compartment by either completing their vaccinations or by becoming infected with COVID-19. It is important to note that when $\omega _i$ is 0 for all age groups, i, the PAIRDV-Virulence model is identical to the SIRDV-Virulence model, where the COVID-19 ‘Pro Vaxxer’ compartment of the PAIRDV-virulence model acts as the Susceptible compartment of the SIRDV-Virulence model.

As shown in Fig. 5b, an increase in the proportion of Anti/Non-Vaxxers will lead to higher virulence and in turn a higher number of infected cases and deaths. The higher the fraction of vaccinated people, the lesser will be the number of deaths and infected cases due to a lower virulence. The future simulations were run in sets, first varying $\omega _i$ for each age group while keeping that of the other age groups constant. For these simulations, when $\omega _i$ was varied for a single age group, *i*, the dynamics of age group *i* had significant changes, but negligible changes in the dynamics of other age groups were observed. For the second set of simulations, $\omega _i$ was varied for all three age groups simultaneously. These results are shown in Fig. 5c–h. Based on these simulations, an increase in $\omega _i$ will result in an increase in cases (Fig. 5c–e) and deaths (Fig. 5f–h) for all three age groups. The proportion of anti-vaxxers affects the children and adults more than the seniors, the reason being a large fraction of seniors had already been vaccinated by the start of the simulated prediction time period.

### Effect of reinfection

We studied the effect reinfection using a modified compartmental model, which has a transition term from the recovered to the infected compartment and a reinfection factor. The modified model was abstracted as its dimensionless form:

8 $$\begin{aligned}&\frac{dx_{S,i}}{d\tau } = -x_{S,i}y K_i-a_ix_{S,i}\;, \end{aligned}$$

9 $$\begin{aligned}&\frac{dx_{I,i}}{d\tau } = x_{S,i}y K_i+\sigma x_{V,i}y K_i-b_ix_{I,i}-c_ix_{I,i}+f_ix_{R,i}y K_i\;, \end{aligned}$$

10 $$\begin{aligned}&\frac{dx_{R,i}}{d\tau } = b_ix_{I,i}-f_ix_{R,i}y K_i\;, \end{aligned}$$

11 $$\begin{aligned}&\frac{dx_{D,i}}{d\tau } = c_ix_{I,i}\;, \end{aligned}$$

12 $$\begin{aligned}&\frac{dx_{V,i}}{d\tau } = a_ix_{S,i}-\sigma x_{V,i}y K_i\;, \end{aligned}$$

13 $$\begin{aligned}&\frac{dy}{d\tau } = \sum ^{3}_{i=1}d_ix_{I,i}-ey\;, \end{aligned}$$

where $f_i$ is the reinfection parameter accounting for the fraction of recovered people who can be reinfected, and all other dimensionless parameters are defined in Table 1. To test the effect of reinfection on the COVID-19 dynamics, we test varying values of $f_i$ in our prediction simulations.

**Table 1:** Definition of variable for the dimensionless SIRDV-Virulence model.

Dimensionless variables		
Variable	Mathematical expression	Significance
${x_{S,i}}$	${S_i}$/N	*Susceptible fraction*
${x_{I,i}}$	${I_i}$/N	*Infected fraction*
${x_{R,i}}$	${R_i}$/N	*Recovered fraction*
${x_{D,i}}$	${D_i}$/N	*Fraction dead*
${x_{V,i}}$	${V_i}$/N	*Vaccinated fraction*
*y*	$\mathscr {V}$/${\mathscr {V}_0}$	*Viral multiplication*
${\tau }$	${\beta _0}$ ${\mathscr {V}_0}$t	$\frac{Actual \,\,\, time}{Time \,\,\, to \,\,\, infect \,\,\, {an \,\,\, individual}}$
${a_i}$	${\alpha _i}$ / ${\beta _0}$ ${\mathscr {V}_0}$	$\frac{Time \,\,\,\, to \,\,\, infect \,\,\, an \,\,\, individual}{Time \,\,\,\, to \,\,\,\, vaccinate \,\,\,\, an \,\,\,\, individual}$
${b_i}$	${\gamma _i}$(1-${\mu _i}$) / ${\beta _0}$ ${\mathscr {V}_0}$	$\frac{Time \,\,\,\, to \,\,\, infect \,\,\, an \,\,\, individual}{Weighted \,\,\,\, recovery \,\,\,\, time \,\,\,\, for \,\,\,\, individual}$
${c_i}$	${\rho _i}$ ${\mu _i}$ / ${\beta _0}$ ${\mathscr {V}_0}$	$\frac{Time \,\,\,\, to \,\,\, infect \,\,\, an \,\,\, individual}{Weighted \,\,\,\, mortality \,\,\,\, time \,\,\,\, for \,\,\,\, individual}$
${d_i}$	${k_{V,i}}$N / ${\beta _0}$ ${\mathscr {V}_0}^{2}$	*Time to infect an individual* $\times$ *Rate of viral multiplication relative to* ${\mathscr {V}_0}$ *and* *N*
*e*	${k^{'}_v}$ / ${\beta _0}$ ${\mathscr {V}_0}$	*Time to infect an individual* $\times$ *Rate of viral death*
${K_i}$	${\beta _i}$ / ${\beta _0}$	*Relative dimensionless transmissibility*
${\sigma _i}$	${\sigma _i}$	*Vaccine inefficacy*

**Figure 6:**
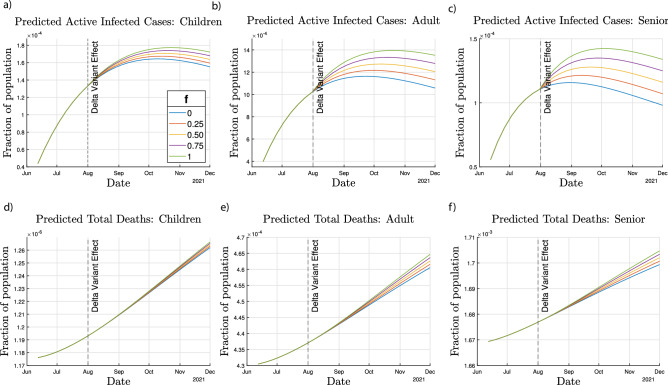
COVID-19 Reinfection Effect. Varying the reinfection parameter, $f_i$, the predicted cases and deaths are shown for (**c**, **f**) seniors, (**b**, **e**) adults, and (**a**, **d**) children as a result of simultaneously changing $f_i$ for each age group.

We simulated the future infections and total deaths for 5 different values of $f_1=f_2=f_3$ ranging from 0 (no reinfection) to 1 (entire recovered population being susceptible to reinfection). As seen in Fig. 6, as the value of $f_i$ increases, the number of active infections increases. The increase in deaths with increasing $f_i$ however is less significant than that for infections.

## Discussion

The conducted simulations, which predict COVID-19 dynamics starting in August 2021, indicated that in the case of increased transmission and decreased vaccine efficacy as a result of the Delta variant, there would be a resurgence in COVID-19 active infected cases and an increase in the total COVID-19 deaths. Among the three age groups considered, the dynamics of the adult and children age groups are more sensitive to the changes introduced by the Delta variant. This is justified by the fact that a large fraction of the senior population had already either been infected or vaccinated by the end of July 2021, relative to the other two age groups.

In the case of an unchanged transmission rate of the virus and unchanged vaccine efficacy (maintaining the same values from the fourth fitted time period), the peak active infections were expected to occur in October 2021. In the worst case scenario simulated (transmission rate, $K_i$, doubled and the vaccine efficacy, $(1-\sigma )=0.8$), the number of active infections at the peak is expected nearly double and occur in December 2021. Although there will be a slight increase in total deaths in this scenario, it will not be a significant one.

From the simulations we saw that both the active infections and the total deaths were more strongly dependent on transmission rate of the virus as opposed to the vaccine efficacy. This is justified by the structure of our model, which reflects the true nature of the viral transmission. For a reduced vaccine efficacy, the transition from the vaccinated compartment to the infected compartment will increase. However, for an increased transmissibility, transition from both the susceptible and vaccinated compartments to the infected compartment will increase.

Three sets of heat maps (Fig. 4) were created to evaluate three different vaccination scenarios: priority to seniors, priority to adults, and priority to children. These maps show that the total COVID-19 deaths infections were heavily dependent on both relative dimensionless $a_1$ (children) and $a_2$ (adults) as compared to $a_{3}$ (seniors). These conclusions can be explained by the fact that a large fraction of adults and children were still left to be vaccinated at the end of July 2021, and any changes in vaccination rate for these two age groups would have a greater effect on the infections, deaths, and completed vaccinations for the overall population. Further, the children do not contribute significantly to the total COVID-19 deaths due to very low mortality rate; hence, changes in $a_1$ won’t significantly affect the total deaths which are heavily dominated by adults and seniors. Since the adult age group consists of the greatest proportion of the US population, any change in vaccination rate for adults will have a drastic effect on the overall dynamics. Thus, it can be concluded from the heat maps that prioritizing the adults and children over seniors for vaccination will be a more effective approach for minimizing the future infected cases and deaths and maximizing the fraction of the vaccinated population.

The fraction of Anti/Non-Vaxxers had a considerable effect on the active infected cases and total deaths, especially in the children and adult age groups. For the seniors the effect was not as pronounced, primarily due to the fact that a large fraction of the senior population had either already been infected or vaccinated by the end of July 2021. For $\omega _2 = 0.5$ (half the susceptible adult population at the beginning of the future simulated time period being Anti/Non-Vaxxers), the total death increases by approximately 6${\%}$ (compared to $\omega _2 = 0$) and causes a delay in the decline of active infected cases. A similar effect is visible in the children age group where an increase of around 3.7${\%}$ is seen in the total deaths for $\omega _1 = 0.5$ (compared to $\omega _1 = 0$). The temporal delay in reduction of active cases in the children age group due to a high proportion of Anti/Non-Vaxxers is longer compared to the other age groups.

The reinfection of the recovered population could cause changes in the dynamics of the pandemic, as seen from the results in Fig. 6. For the children age group, the total deaths at the end of simulated time period for the worst case scenario ($f=1$) is higher than the no reinfection case ($f=0$) by 3.7%, whereas for adults and seniors, this value is higher by 9.3% and 3.3%, respectively. Further, there are changes in the active number of cases across all three age groups in the case of reinfection. As seen in Fig. 6, the active infected cases at the peak in the worst case scenario ($f=1$) for the children, adult and senior age groups are higher than the cases in case of no reinfection ($f=0$) by 7.9%, 20%, and 23%, respectively.

While our simulations focus on the dynamic effects of the Delta variant, our model can also be applied to the other strains of COVID-19. The parameters *K* (relative dimensionless transmissibility), *c* (relative dimensionless mortality 1), and $\sigma$ (vaccine inefficacy) can change for the different variants of concern: Alpha, Beta, Gamma, Delta, Omicron, and other future variants. For example, since the Omicron variant has been reported to be more transmissible and less deadly than the Delta one^49,50^, its effects could be simulated using a higher *K* value and a lower *c*, relative to those values simulated for Delta. Regarding the vaccine inefficacy, some studies have reported that vaccinations provide no protection against symptomatic disease caused by the Omicron variant 20 to 24 weeks after a second dose^51^. This can be captured by an increased value of $\sigma$ in our model. The ability to tune the model parameters per the properties of COVID-19 strains adds flexibility and robustness to the model.

## Conclusion

Due to the continuously evolving nature of the COVID-19 pandemic, it is important to understand what causes the changing dynamics in order to predict its future behavior. In this study, we select four factors that we identify as essential to understanding and predicting COVID-19 dynamics: (1) effect of SARS-CoV-2 virus mutants, (2) effect of vaccine allocation and rollout speed, (3) effect of Anti/Non-Vaxxers, (4) effect of reinfection. In addition to these factors, we simultaneously study the influence of age groups on COVID-19 dynamics. We create a novel compartmental model, which is stratified by age groups (children, adult, and senior) and simulates infection using a virulence environment derived using population balance equations.

The Delta variant is simulated by increasing COVID-19 transmissibility and decreasing vaccine efficacy. Our predictions provide estimates of the peak magnitude and time of infection for varying scenarios, which helps to prepare the healthcare system.

To study the dynamics associated with vaccine allocation and rollout speed, we use fitted data to make practical predictions of COVID-19 infections and deaths as a result of prioritizing each age group and varying the relative speed at which vaccines are administered. The simulation results suggest that the optimum vaccination strategy is to prioritize the children and adult age groups, whose dynamics are shown to be more sensitive to vaccine administration.

The model is modified to study the effect of people who are unwilling and/or unable to receive a COVID-19 vaccination. As the simulations results indicate, a larger proportion of Anti/Non-Vaxxers will result in a larger number infections and deaths, especially in the adult and children populations. Additionally, a large fraction of unvaccinated people could result in a resurgence of the pandemic.

To study the effect of waning immunity of recovered individuals, the reinfection parameter of a modified model is varied. Reinfection can cause a significant increase in the active infected cases, but the changes in total death may be relatively less significant. As seen for the worst case scenario simulated, a high reinfection rate of recovered individuals can lead to a resurgence of the pandemic.

## Methods

Our model considers the interactions among different age groups using population balance equations^39,40^. In the following sections, we start with the introduction of the compartmental model and the model for the infection transmission among three different age groups (children, adults, and seniors). We then use the population balance model to derive the average equations, which can be converted to dimensionless dynamic equations with characteristic quantities (dimensionless numbers). We fit the model by quantifying these dimensionless numbers using Centers for Disease Control and Prevention (CDC) released infection, death, and vaccination data. These quantities are then used to make predictions.

### Compartmental model

 As shown in Fig. 1b, our SIRDV-Virulence ( Susceptible-Infected-Recovered-Dead-Vaccinated-Virulence) model consists of 6 compartments: Susceptible ($S_i$), Infected ($I_i$), Recovered ($R_i$), Dead ($D_i$), Vaccinated ($V_i$), and Virulence ($\mathscr {V}$), where *i* denotes different age groups (1: children, 2: adult, and 3: senior). To derive the inter-compartmental and inter-age group interactions, we start by characterizing the average infection transfer among different age groups.

### Average infection inter-transfer frequency between any two age groups 

 Here, we identify the age-specific infection transfer frequency as $q(a_g,a_{g}';t)dt$, which is the probability that individuals from two age groups $a_g$ and $a_g'$ will be in close proximity during the time interval *t* to $t+dt$. For instance, we choose $a_g$ to be in the children range denoted as the population $g=S_1$ (*S*: Susceptible) and $a_g'$ to be any other age range (e.g. adult, senior) identified as $g'\in \{I_2,~I_3\}$ or the same age range $g'=I_1$ (*I*: Infected). The average meeting frequency between individuals in *g* and $g'$ is identified as:

14 $$\begin{aligned} \bar{q}_{g-g'}(t) = \frac{\int _g\int _{g'} da_g da_g' g(a_g, t) g'(a_g', t)q(a_g,a_g';t)}{\int _sda_g g(a_g, t)\int _{s'}da_g' g'(a_g', t)}= \frac{\int _g\int _{g'} da_g da_g' g(a_g, t) g'(a_g', t)q(a_g,a_g';t)}{\bar{g}(t)\bar{g}'(t)}. \end{aligned}$$

### Population balance model of infection dynamics

 A model considers the transfer of infection among the children, adult, and senior age groups. Note that this model can be extended to any number of groups. First we must add two internal coordinates where *v* denotes the viral population in the individual and $a^r$ represents the age range of the population. Thus, we let $\phi (v,a^r,t)dvda^r$ be the number of individuals with a viral infection in the range *v* to $v+dv$ and an age range of $a^r$ to $a^r+da^r$. The viral infection in each individual changes at the averaged rate $\bar{\dot{\mathscr {V}}}_i$ in population *i*, where $\mathscr {V}(t)=\int _vdv\int da^r v\phi (v,a^r,t)$ is denoted as virulence. Note that in this work, we focus on the effect of the virulence environment among human interactions and neglect the factor of viruses resident on solid surfaces and droplets^52^. Therefore, this definition of virulence, $\mathscr {V}$, excludes viruses resident on the solid surfaces in the environment, which can also cause viral spread to the extent that people come into contact with infected surfaces.

We note the total population of infected individuals $I_i$,

15 $$\begin{aligned} I_i = \int dv \int _{a^r_i} da^r \phi (v,a^r,t),\quad i=1,2,3 \end{aligned}$$

where $a^r_i$ indicates the age range of group *i*; we assume that a freshly infected individual has $v=0$. Then, $\bar{\dot{\mathscr {V}}}_{i}(v)$ is the viral infection rate of change in the individual. An infected individual with viral intensity *v* may die with the frequency $k_{d}(v,a^r)$ or recover with frequency $k_{r}(v,a^r)$. The population balance equation for the diseased population is given by

16 $$\begin{aligned}&\frac{\partial \phi (v,a^r,t)}{\partial t} + \frac{\partial }{\partial v}\left[ \bar{\dot{\mathscr {V}}}(v)\phi \right] = -[k_{r}(v,a^r)+k_{d}(v,a^r)]\phi . \end{aligned}$$

We denote the existence of probability densities $p_i(v)$ in age group *i* for developing an infection on contact with an infected individual of viral density *v*, $\int _o^{\infty }p_idv=1$. The boundary condition for equation (16) is

17 $$\begin{aligned} \int _{a^r_i}da^r~\phi (0,a^r,t)\bar{\dot{\mathscr {V}}}(0)= & {} S_i(t) \int _{a^r_i}da^r\int _o^{\infty }dv\sum _{j=1}^3[\bar{q}_{S_i-I_j}(t)\phi _{j}(v,a^r,t)]p_{i}(v)\nonumber \\&+V_{i}(t)\int _{a^r_i}da^r \int _o^{\infty }dv\sum _{j=1}^3[\bar{q}_{V_i-I_j}(t)\phi _{j}(v,a^r,t)]p_{i}(v), \end{aligned}$$

where $S_i$ is the susceptible population, $V_i$ is the vaccinated population, and $\bar{q}_{S_i-I_j}$ is the meeting frequency between susceptible $S_i$ and infected $I_j$.

### Average models

 We further observe that the total infection equation obtained by integrating equation (16) with respect to *v* from zero to infinity and *a* within the age range ($a_i$),

18 $$\begin{aligned} \frac{dI_{i}}{dt}= & {} \mathscr {V}S_i\beta _i+\mathscr {V}V_i\sigma \beta _i-(1-\mu _i)\gamma _iI_i-\mu _i\rho _iI_i, \end{aligned}$$

where we applied the boundary condition equation (17). The transmission rate, $\beta _i$, and vaccine inefficacy, $\sigma _i$, are thus defined as

19 $$\begin{aligned} \beta _i=\frac{\int _{a^r_i}da^r\int _o^{\infty }dv\sum _{j=1}^3[\bar{q}_{S_i-I_j}(t)\phi _{j}(v,a^r,t)]p_{i}(v)}{\int _o^{\infty }da^r\int _o^{\infty }dv~v \phi (v,a^r,t)}, \end{aligned}$$

20 $$\begin{aligned} \sigma _i=\frac{\int _{a^r_i}da^r\int _o^{\infty }dv\sum _{j=1}^3[\bar{q}_{V_i-I_j}(t)\phi _{j}(v,a^r,t)]p_{i}(v)}{\int _{a^r_i}da^r\int _o^{\infty }dv\sum _{j=1}^3[\bar{q}_{S_i-I_j}(t)\phi _{j}(v,a^r,t)]p_{i}(v)}. \end{aligned}$$

The rate constant, $\beta$, characterizes the extent of the inter-transmission among and between different age groups, and $\sigma$ measures the ratio of transmission rate between vaccinated and susceptible populations. The death and recovery rates are expressed as

21 $$\begin{aligned} \mu _i\rho _i=\frac{\int dv\int _{a^r_i} da^r k_{d}(v,a^r)\phi (v,a^r,t)}{I_i}, \end{aligned}$$

22 $$\begin{aligned} (1-\mu _i)\gamma _i=\frac{\int dv \int _{a^r_i} da^r k_{r}(v,a^r)\phi (v,a^r,t)}{I_i}. \end{aligned}$$

Next, we multiply equation (16) by *v* and integrate with respect to *v* and $a^r$ from 0 to infinity:

23 $$\begin{aligned} \frac{d\mathscr {V}}{dt}=\sum ^{3}_{i=1}(k_{v,i}I_i)-k'_v\mathscr {V}, \end{aligned}$$

where

24 $$\begin{aligned} k_{v,i} = \frac{\int _{a^r_i}da^r\int _0^{\infty }dv\bar{\dot{\mathscr {V}}}\phi }{\int dv \int _{a^r_i} da^r \phi },\quad k'_v = \frac{\int _{a^r_i}da^r\int _0^{\infty }dv(k_r+k_d)\phi }{\int vdv \int _{a^r_i} da^r \phi }. \end{aligned}$$

Given the dynamics of virulence, $\mathscr {V}$, and infection, $I_i$, the derivation of the equations for the remaining compartments is straightforward. These equations have been displayed in the supplementary material.

### Dynamics models

To understand the dynamics of both the viral and human populations, the modeling of the system in a considered geometric domain was abstracted as its dimensionless form:

25 $$\begin{aligned}&\frac{dx_{S,i}}{d\tau } = -x_{S,i}y K_i-a_ix_{S,i}\;, \end{aligned}$$

26 $$\begin{aligned}&\frac{dx_{I,i}}{d\tau } = x_{S,i}y K_i+\sigma x_{V,i}y K_i-b_ix_{I,i}-c_ix_{I,i}\;, \end{aligned}$$

27 $$\begin{aligned}&\frac{dx_{R,i}}{d\tau } = b_ix_{I,i}\;, \end{aligned}$$

28 $$\begin{aligned}&\frac{dx_{D,i}}{d\tau } = c_ix_{I,i}\;, \end{aligned}$$

29 $$\begin{aligned}&\frac{dx_{V,i}}{d\tau } = a_ix_{S,i}-\sigma x_{V,i}y K_i\;, \end{aligned}$$

30 $$\begin{aligned}&\frac{dy}{d\tau } = \sum ^{3}_{i=1}d_ix_{I,i}-ey\;, \end{aligned}$$

where a list of dimensionless parameters is defined in Table 1. As shown in Fig. 1a and Eq. (25), people from the susceptible compartment move into the infected or vaccinated compartment. The transition rate from the susceptible to the infected compartment is characterized by the dimensionless transmissibility of the virus, $K_i$, and the dimensionless viral load, *y*. The dimensionless vaccination rate, $a_i$, determines the rate of transition from the susceptible to the vaccinated compartment. The population in the vaccinated compartment (Eq. (29)) is governed by the vaccination rate, $a_i$, and the vaccine inefficacy^53^, $\sigma _i$, in addition to $K_i$ and *y*. The dynamics of the infected compartment (Eq. 26) are determined by the rates of infection of susceptible and vaccinated populations, as well as by recovery and death rates. From the infected compartment, a person can move into the recovered or death compartment; these transition rates are governed by the dimensionless recovery rate, $b_i$, and the dimensionless mortality rate, $c_i$ (Eqs. (27) and (28), respectively). The growth of virulence is governed by the dimensionless number $d_i$, through an interaction with the infected compartment (Eq. 30). There is a decay term for the virulence dictated by dimensionless number *e*, related to the lifespan of the virus.

### Data processing

We considered three different age groups for analysis: children (12–17 years), adults (18–64 years), and seniors (65 years and older). These age group divisions matched those of the CDC’s vaccination data set^54^. Because COVID-19 infection and death data sets used for this study had more age groups than the vaccination data set, the age group data of the infection and death data sets were combined to form uniform age groups across all three data sets. To analyze the COVID-19 dynamic evolution, we quantified dimensionless parameter values based on weekly reported infections, weekly reported deaths, and daily completed vaccinations. These data sets were normalized by the total population. For the analysis, the total population of the United States was assumed to be 332.5 million^55^, based on the US population at the time of data collection. The United States Census 2019 age distribution^55^ was used to estimate the population of each age group in the United States. Because each data set used in this study had a unique format, the data sorting and processing for each set were different (see supplementary material).

### Simulation scheme

 Before fitting the dynamic SIRDV-Virulence equations (25)–(30) to the data sets, we estimated the initial population value for each compartment (supplementary material).

Since data were not available for the size of the susceptible population, this was found using a population balance once the initial value of each other compartment population was found:

31 $$\begin{aligned} x_{S_i} = N_i/N-(x_{I_i}+x_{R_i}+x_{D_i}+x_{V_i})\;, \end{aligned}$$

where $N_i$ is the population of age group *i* and *N* is the total population (ages 12 and older). For simplicity, it is assumed that the population of each age group is constant during the time period for fitting the model and completing the future predictions. This constant population includes people that have died due to COVID-19 since the death compartment is part of the constant population being studied. The movement into and out of each age group is assumed to be equal. For seniors, the number of people ageing into the population is assumed to be equal to deaths from causes other than COVID-19.

We non-dimensionalize the data so it is consistent with the dimensionless equations used in the model. When fitting the model to individual age group dynamics, data were normalized with the population of each age group. When fitting the model for the entire population, the data were normalized using the total United States population (ages 12+).

A segmented fitting method was used to fit the derived model (Results). This method has been previously used in other COVID-19 studies. For example, Tian et al modeled the early pandemic in mainland China by dividing the time period into three parts: exponential growth, crossover, and final descent^21^. This segmentation approach is particularly suitable when the infection dynamics are different in each time period and the parameters fitted for each period give physical insight into various factors; the insight gained can aid in choosing parameter values for predictions. The segmented fitting method adds robustness to our model by producing a value for each parameter during each time period with differing dynamics, rather than generating a single value for each parameter that would not provide insight into the differing dynamics characteristic of each time period.

## Supplementary Information

Supplementary Information.

## Data Availability

The data and code that support the findings of this study are available from the corresponding authors. For infection data, COVID-19 weekly cases by age group were collected from the Centers for Disease Control and Prevention (CDC) Data Tracker’s “COVID-19 Weekly Cases per 100,000 Population by Age, Race/Ethnicity, and Sex” data visualization at https://covid.cdc.gov/covid-data-tracker/#demographicsovertime. For each week, data were manually collected for each age group and stored in a csv file. COVID-19 weekly deaths by age group were collected from the CDC dataset, “Provisional COVID-19 Deaths by Week, Sex, and Age” at https://data.cdc.gov/NCHS/Provisional-COVID-19-Deaths-by-Week-Sex-and-Age/vsak-wrfu. The daily cumulative completed vaccinations and daily cumulative administered vaccinations were collected from the CDC dataset, “COVID-19 Vaccinations in the United States, Jurisdiction” at https://data.cdc.gov/Vaccinations/COVID-19-Vaccinations-in-the-United-States-Jurisdi/unsk-b7fc. Population data for the United States, including total population and age distribution, were collected from the United States Census’s “U.S. and World Population Clock” at https://www.census.gov/popclock/.
